# Supplementary material for: Assessment of the Impact of Dietary Supplementation with Epigallocatechin Gallate (EGCG) on Antioxidant Status, Immune Response, and Intestinal Microbiota in Post-Weaning Rabbits
Source: Animals (Basel). 2024 Oct 18;14(20):3011. doi: 10.3390/ani14203011 (PMC11504044; doi:10.3390/ani14203011)
Supplement: Supplementary file 1 [file animals-14-03011-s001.zip › animals-3239994-supplementary.pdf]

Table S1. ANOSIM analysis between EGCG treated groups and the control group[1)]

Comparison	Permutations	R	*P*-value
A vs B	200	0.0593	0.2687
A vs C	200	0.0611	0.2438
A vs D	200	0.4648	0.0199
A vs E	200	0.470	0.0050
A vs F	200	0.6000	0.0149

[1)] A the control group; B 200mg/kg EGCG treated group; C 400mg/kg EGCG treated group; D 600mg/kg EGCG treated group; E 800mg/kg EGCG treated group; F 1000mg/kg EGCG treated group.

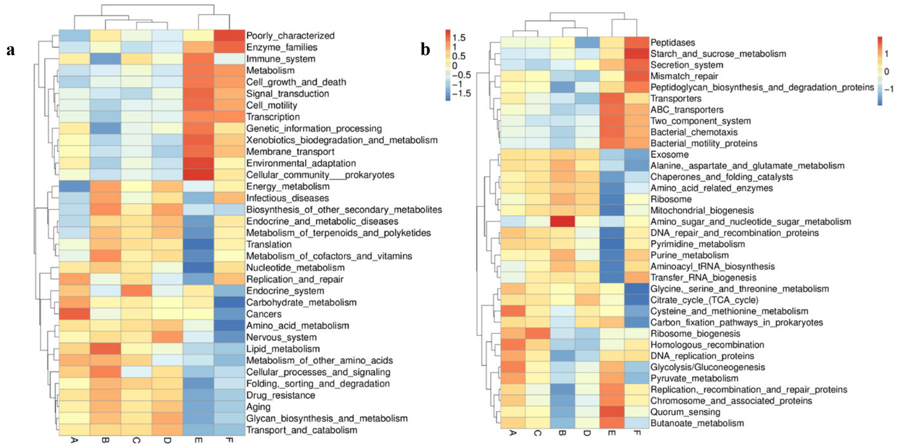

Figure S1. The heatmap of predicted function of microbiota in rabbits fed with different dosage of EGCG based on KEGG level 2 (**a**) and level 3(**b**). A the control group; B 200mg/kg EGCG treated group; C 400mg/kg EGCG treated group; D 600mg/kg EGCG treated group; E 800mg/kg EGCG treated group; F 1000mg/kg EGCG treated group.
